# ICOS-Fc as innovative immunomodulatory approach to counteract inflammation and organ injury in sepsis

**DOI:** 10.3389/fimmu.2022.992614

**Published:** 2022-09-02

**Authors:** Gustavo Ferreira Alves, Ian Stoppa, Eleonora Aimaretti, Chiara Monge, Raffaella Mastrocola, Elisa Porchietto, Giacomo Einaudi, Debora Collotta, Ilaria Bertocchi, Elena Boggio, Casimiro Luca Gigliotti, Nausicaa Clemente, Manuela Aragno, Daniel Fernandes, Carlo Cifani, Christoph Thiemermann, Chiara Dianzani, Umberto Dianzani, Massimo Collino

**Affiliations:** ^1^ Department of Neurosciences (Rita Levi Montalcini), University of Turin, Turin, Italy; ^2^ Department of Health Sciences, Università del Piemonte Orientale, Novara, Italy; ^3^ Department of Clinical and Biological Sciences, University of Turin, Turin, Italy; ^4^ Department of Drug Science and Technology, University of Turin, Turin, Italy; ^5^ Pharmacology Unit, School of Pharmacy, University of Camerino, Camerino, Italy; ^6^ Department of Pharmacology, Federal University of Santa Catarina, Florianópolis, Brazil; ^7^ William Harvey Research Institute, Bart’s and The London School of Medicine and Dentistry, Queen Mary University of London, London, United Kingdom

**Keywords:** sepsis, inflammation, ICOS (inducible co-stimulatory molecule), cecal ligation and puncture, osteopontin (OPN)

## Abstract

Inducible T cell co-stimulator (ICOS), an immune checkpoint protein expressed on activated T cells and its unique ligand, ICOSL, which is expressed on antigen-presenting cells and non-hematopoietic cells, have been extensively investigated in the immune response. Recent findings showed that a soluble recombinant form of ICOS (ICOS-Fc) can act as an innovative immunomodulatory drug as both antagonist of ICOS and agonist of ICOSL, modulating cytokine release and cell migration to inflamed tissues. Although the ICOS-ICOSL pathway has been poorly investigated in the septic context, a few studies have reported that septic patients have reduced ICOS expression in whole blood and increased serum levels of osteopontin (OPN), that is another ligand of ICOSL. Thus, we investigated the pathological role of the ICOS-ICOSL axis in the context of sepsis and the potential protective effects of its immunomodulation by administering ICOS-Fc in a murine model of sepsis. Polymicrobial sepsis was induced by cecal ligation and puncture (CLP) in five-month-old male wild-type (WT) C57BL/6, ICOS^-/-^, ICOSL^-/-^ and OPN^-/-^ mice. One hour after the surgical procedure, either CLP or Sham (control) mice were randomly assigned to receive once ICOS-Fc, ^F119S^ICOS-Fc, a mutated form uncapable to bind ICOSL, or vehicle intravenously. Organs and plasma were collected 24 h after surgery for analyses. When compared to Sham mice, WT mice that underwent CLP developed within 24 h a higher clinical severity score, a reduced body temperature, an increase in plasma cytokines (TNF-α, IL-1β, IL-6, IFN-γ and IL-10), liver injury (AST and ALT) and kidney (creatinine and urea) dysfunction. Administration of ICOS-Fc to WT CLP mice reduced all of these abnormalities caused by sepsis. Similar beneficial effects were not seen in CLP-mice treated with ^F119S^ICOS-Fc. Treatment of CLP-mice with ICOS-Fc also attenuated the sepsis-induced local activation of FAK, P38 MAPK and NLRP3 inflammasome. ICOS-Fc seemed to act at both sides of the ICOS-ICOSL interaction, as the protective effect was lost in septic knockout mice for the ICOS or ICOSL genes, whereas it was maintained in OPN knockout mice. Collectively, our data show the beneficial effects of pharmacological modulation of the ICOS-ICOSL pathway in counteracting the sepsis-induced inflammation and organ dysfunction.

## Introduction

Sepsis is a life-threatening medical emergency characterized by a complex interplay of pro- and anti-inflammatory host responses, resulting in multiple organ dysfunction that can ultimately lead to death (1). Currently, deaths from sepsis correspond to nearly 20% of all deaths worldwide, and there is still no specific treatment available (2). The inducible T cell co-stimulator (ICOS, also known as CD278) belongs to the CD28 family of co-stimulatory immunoreceptors. It is a type I transmembrane glycoprotein whose expression is rapidly upregulated upon T cells activation (3). ICOS binds to its unique ligand (ICOSL, also known as CD275 or B7h), a member of the B7 family highly expressed on antigen-presenting cells (APCs) and non-hematopoietic cells under inflammatory stimuli (4–5). Thus far, the role of ICOS-ICOSL interaction has been poorly investigated in sepsis, although recent findings report that ICOS expression is reduced in whole blood of septic patients (6), and that reduced ICOS levels are strongly associated with organ dysfunction (7). To date, it is very well documented that the ICOS-ICOSL axis may display bidirectional effects. On the one hand, ICOS triggering modulates cytokine production in activated T cells and contributes to T regulatory (Treg) cells differentiation and survival (8–9). Given the fact that both animals and septic patients have an increased percentage of circulating Treg cells (10–12), it is suggestive that ICOS triggering may play a role in the septic immunosuppressive status. On the other hand, ICOSL triggering by ICOS may exert anti-inflammatory effects *via* responses, such as modulating the maturation and migration of macrophage and dendritic cells and the endothelial cell adhesiveness (13).

Recently, another ligand for ICOSL has been identified, osteopontin (OPN), an inflammatory mediator that binds to ICOSL in an alternative binding domain to that used by ICOS. Intriguingly, ICOS and OPN exert different and often opposite effects upon ICOSL triggering since OPN stimulates, whereas ICOS inhibits, migration of several cell types and tumor angiogenesis (14–16). Conventionally, a soluble recombinant form of ICOS (ICOS-Fc) has been designed by fusing a cloned extracellular portion of human or mouse ICOS with an Fc IgG1 portion and this molecule has been shown to trigger ICOSL thus promoting down-stream responses (17).

*In vitro*, ICOS-Fc inhibits adhesiveness of endothelial cells toward polymorphonuclear cells and tumor cells and migration of endothelial cells and tumor cells (15). These ICOS-Fc effects can also be recorded in dendritic cells (DC), along with modulated cytokine release and antigen cross-presentation in class I major histocompatibility complex molecules (13), while in osteoclasts, ICOS-Fc inhibits differentiation and function (18). *In vivo*, ICOS-Fc inhibits tumor growth and metastasis, development of osteoporosis, liver damage induced by acute inflammation following treatment with CCl4, and it favors skin wound healing (18–21). Nevertheless, little is known about the molecular mechanism(s) involved in ICOSL-mediated inflammatory response. The p38 MAPK, a well-known mediator that drives inflammation through upregulation of several pro-inflammatory cytokines such as TNF-α and IL-6 (22), and the NOD-like receptor protein 3 (NLRP3) inflammasome, able to induce the release of IL-1β and IL-18 and promote cell death by pyroptosis (23), are two of the most well characterized signaling pathways involved in the activation of the cytokine storm that contributes to organ dysfunction during sepsis. Furthermore, their pharmacological or genetic inhibition has been shown to reduce sepsis-related mortality (22–24). Finally, a non-receptor protein kinase namely Focal adhesion kinase (FAK) has been recently reported to signal inflammation downstream of the Toll-like receptor 4 upon lipopolysaccharide (LPS) challenge in macrophages and lung tissues (25). Therefore, here we investigated, for the first time, the pathological role of ICOS-ICOSL axis in the context of sepsis, its impact on selective inflammatory pathways and the potential protective effects of its immunomodulation by administering ICOS-Fc in an experimental model of sepsis.

## Material and methods

### Animals and ethical statement

Inbred wild-type (WT, C57BL/6) mice, ICOSL knockout mice (ICOSL^-/-^, B6.129P2-*Icosl* ^tm1Mak^/J), ICOS knockout mice (ICOS^-/-^, B6.129P2-*Icos* ^tm1Mak^/J) and OPN knockout mice (OPN^-/-^, B6.129S6(Cg)-*Spp1* ^tm1Blh^/J) were purchased from Envigo laboratories, (IT) and The Jackson Laboratory (Bar Harbor, ME, USA). Mice were housed under standard laboratory conditions, such as room temperature (25 ± 2°C) and light-controlled with free access to water and rodent chow for four weeks prior starting the experimental procedures. All animal protocols reported in this study followed the ARRIVE guidelines (26) and the recommendations for preclinical studies of sepsis provided by the MQTiPSS (27) The procedures were approved by the University’s Institutional Ethics Committee as well as the National Authorities (Protocol number: 855/2021).

### Cecal ligation and puncture (CLP)-induced sepsis model

Polymicrobial sepsis was carried out by CLP surgery in male, five-month-old mice. Mice were initially placed in an anesthetisia chamber (3% isoflurane -IsoFlo, Abbott Laboratories – delivered in oxygen 0.4 L/min), then kept under anaesthesia throughout surgery with 2% isoflurane delivered in oxygen 0.4 L/min *via* a nosecone. The body temperature was maintained at 37 °C through a homoeothermic blanket and constantly monitored by a rectal thermometer. Briefly, a mid-line laparotomy (~1.0 cm) was performed in the abdomen, exposing the cecum. The cecum was then totally ligated just below the ileocecal valve and a G-21 needle was used to puncture the ligated cecum in a single through-and-through manner. A small amount (droplet, ~3mm) of fecal content was released from the cecum which was carefully relocated into the peritoneum. Sham mice underwent the same surgical procedure, but without CLP. All animals received Carprofen (5 mg/kg, s.c.) as an analgesic agent and resuscitation fluid (0.9% NaCl, 50 mL/kg, s.c.) at 37°C. Mice were constantly monitored post-surgical and then placed back into fresh clean cages.

At 24 h, body temperature and a clinical score to assess symptoms consistent with murine sepsis were recorded blindly. The following 6 criteria were used for the clinical score: lethargy, piloerection, tremors, periorbital exudates, respiratory distress and diarrhea. An observed clinical score >3 was considered as severe sepsis, while a score between 3 and 1 was considered as moderate sepsis (28).

### Study design

**Figure 1:**
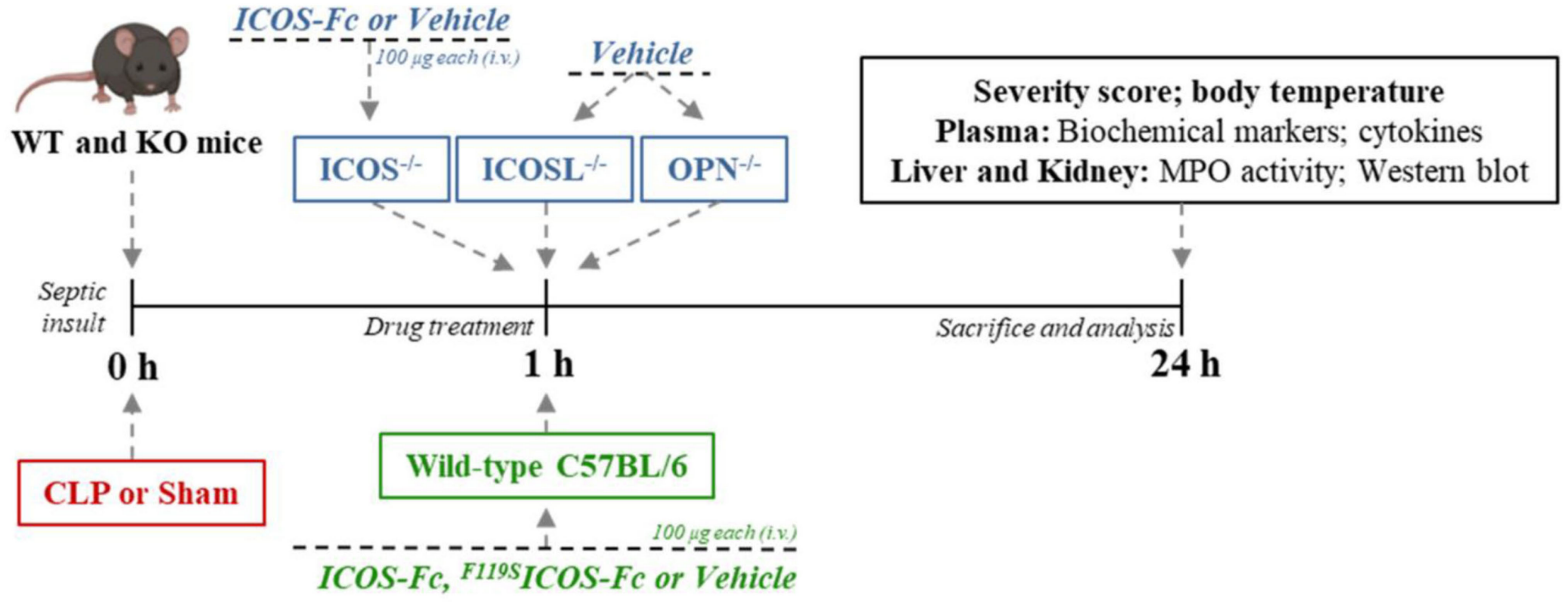
Timeline of the experimental design to investigate the role of ICOS-Fc in sepsis. Wild-type mice and/or ICOSL, ICOS and OPN knockout mice were randomly selected to undergo either Sham or CLP surgery. One hour later, mice received once either Vehicle (PBS, 100 µL), ICOS-Fc (100 µg) or ^F119S^ICOS-Fc (100 µg) intravenously. At 24 h all parameters were analyzed.

Seventy-two mice were randomized into eight groups (9 mice per group): Sham + Vehicle, CLP + Vehicle, CLP + ICOS-Fc, CLP + ^F119S^ICOS-Fc, CLP ICOSL^-/-^ + Vehicle, CLP ICOS^-/-^ + Vehicle, CLP ICOS^-/-^ + ICOS-Fc and OPN^-/-^ + Vehicle. Treatment was given once one hour after surgery, where mice received either ICOS-Fc (100 µg each), ^F119S^ICOS-Fc (100 µg each) or Vehicle (PBS, pH 7.4, 100 µl each) by intravenous injection (**Figure 1**).

### Blood collection and organ harvesting

Twenty-four h after surgery all mice were anesthetized with isoflurane (3%) delivered in oxygen (0.4 L/min) and euthanized by cardiac exsanguination. Whole blood was withdrawn from each mouse in vials (EDTA 17.1 µM/mL) and plasma content was obtained after centrifugation (13,000 *g*, 10 min at R.T.). Organ samples (liver and kidney) were harvested and placed in cryotubes which were snap frozen in liquid nitrogen for storage at freezer -80°C. The samples were then analyzed in a blinded fashion (**Figure 1**).

### Biomarkers of organ injury and systemic inflammation

Plasma samples were used to measure systemic levels of aspartate aminotransferase (AST) (#7036) and alanine aminotransferase (ALT) (#7018) (as markers of hepatocellular injury), creatinine (#7075) and urea (#7144) (as markers of renal dysfunction) using colorimetric clinical assay kits (FAR Diagnostics, Verona, Italy) according to the manufacturer’s instructions. Systemic cytokine levels were determined in plasma using the Luminex suspension bead-based multiplexed Bio-Plex Pro™ Mouse Cytokine Th17 Panel A 6-Plex (#M6000007NY) assay (Bio-Rad, Kabelsketal, Germany). The cytokines (IL-1β, IL-6, TNF-α, IFN-γ, IL-17 and IL-10) were measured following the manufacturer’s instructions.

### Myeloperoxidase (MPO) activity analysis

MPO activity analysis was carried out in liver and kidney samples as previously described (29). Tissue samples (~100 mg) were homogenized (1:5 w-v) in 20 mM PBS (pH 7.4) and then centrifuged at 4°C (13,000 g, 10 min). Pellets were resuspended in 500 μL of hexadecyltrimethylammonium bromide buffer (0.5% HTAB in 50 mM PBS, pH 6.0). A second centrifugation at 4°C (13,000 g, 10 min was performed and the supernatants (30 μL) were assessed for MPO activity by measuring spectrophotometrically (650 nm) the H_2_O_2_-dependent oxidation of 3,3′,5,5′-tetramethylbenzidine (TMB). Bicinchoninic acid (BCA) protein assay (Pierce Biotechnology Inc., Rockford, IL, USA) was used to quantify the protein content in the final supernatant. MPO activity was expressed as optical density (O.D.) at 650 nm per mg of protein.

### Western blot analysis

Semi-quantitative immunoblot technique was carried out in hepatic and renal tissue samples as previously described (30). Total proteins were extracted from 50 mg of each tissue and the total content was quantified using BCA protein method following the manufacturer’s instructions. Briefly, total proteins (50 µg/well) were separated by 8 and 10% sodium dodecyl sulphate-polyacrylamide gel electrophoresis (SDS-PAGE) and transferred to a polyvinylidene difluoride (PVDF) membrane, which was then blocked with 5% non-fat dry milk prepared in TBS-T buffer for 1 h at RT, followed by incubation with primary antibodies at the dilution 1:1000., rabbit anti-Thr^180^/anti-Tyr^182^ p38 (Cell Signaling #9211); rabbit anti-total p38 (Cell Signaling #9212); mouse anti-NRLP3 (Adipogen- AG-20B-0014-C100); rabbit anti-Caspase-1 (Cell Signaling #24232); rabbit anti-Tyr^397^ FAK (Cell Signaling #3283); rabbit anti-total FAK (Cell Signaling #3285). The membranes were then incubated with a secondary antibody conjugated with horseradish peroxidase (HRP) at the dilution 1:10000 for 1 h at RT (anti-mouse or anti-rabbit, Cell Signaling #7076 and #7074, respectively). Afterwards, the membranes were stripped and incubated with rabbit anti-β-actin (Cell Signaling #4970). Immune complexes were visualized by chemiluminescence and the densitometric analysis was performed using Bio-Rad Image Lab Software 6.0.1. Results were normalized to sham bands.

### Statistical analysis and data presentation

Sample size was determined on the basis of prior power calculations using G-Power 3.1™ software (31). Data are expressed as dot plots (for each mouse) and as mean ± S.E.M of 9 mice per group. Shapiro-Wilk and Bartlett tests were used to verify data distribution and the homogeneity of variances, respectively. The statistical analysis was performed by one-way ANOVA, followed by Bonferroni’s *post-hoc* test. Data not normally distributed, a non-parametric statistical analysis was applied through Kruskal-Wallis followed by Dunn’s *post hoc*-test as indicated in the figure legends. Statistical significance was set at P < 0.05. Statistical analysis was performed using GraphPad Prism^®^ software version 7.05 (San Diego, California, USA).

### Materials

Unless otherwise stated, all reagents were purchased from the Sigma-Aldrich Company Ltd. (St. Louis, Missouri, USA).

## Results

### ICOS-Fc-mediated immunomodulation attenuates clinical status and organ injury/dysfunction triggered by sepsis

Sepsis was induced by CLP in WT mice treated with vehicle, ICOS-Fc or ^F119S^ICOS-Fc (unable to bind ICOSL) and clinical scores and body temperature were recorded after 24 h. Moreover, sepsis was induced in mice deficient for ICOS, ICOSL, or OPN to assess the role the endogenous molecules of the ICOS/ICOSL/OPN system. Finally, a group of ICOS-deficient mice received ICOS-Fc treatment to evaluate the effect of the drug in the absence of the endogenous ICOS.

**Figure 2:**
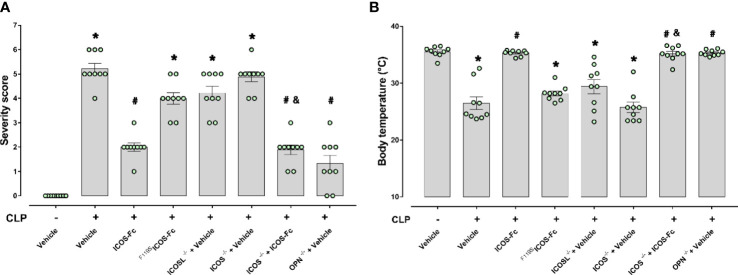
Role of the ICOS-ICOSL axis in the clinical status of experimental sepsis. Wild-type mice and/or ICOSL, ICOS and OPN knockout mice were randomly selected to undergo either Sham or CLP surgery. One hour later, mice received once either Vehicle (PBS, 100 µL), ICOS-Fc (100 µg) or ^F119S^ICOS-Fc (100 µg) intravenously. At 24 h, severity score **(A)** and body temperature **(B)** were recorded. Data are expressed as dot plots (for each animal) and as mean ± S.E.M of 9 mice per group. Severity score was analyzed by a non-parametric test (Kruskal-Wallis) followed by Dunn’s *post hoc*-test, whereas a parametric test (one-way ANOVA) followed by Bonferroni’s *post hoc*-test was used for body temperature. *p<0.05 *vs* Sham + Vehicle; ^#^p<0.05 *vs* CLP + Vehicle; ^&^p<0.05 *vs* ICOS^-/-^ + Vehicle.

Results showed that, as expected, CLP-induced sepsis in WT mice led to a higher clinical severity score (**Figure 2A**) when compared to Sham WT mice, which was also associated with lower body temperature (**Figure 2B**). Intriguingly, treatment with ICOS-Fc improved both clinical score and hyphotermia in WT septic mice, whereas treatment with ^F119S^ICOS-Fc had no effect (**Figures 2A, B**). Analysis of CLP knockout mice showed that ICOS^-/-^ and ICOSL^-/-^ mice showed similar clinical scores and decreased body temperatures as WT mice, whereas OPN^-/-^ mice developed milder sepsis, with lower clinical scores and higher body temperature than WT mice. In ICOS^-/-^ mice, treatment with ICOS-Fc induced similar positive effects as in WT mice (**Figures 2A, B**).

**Figure 3:**
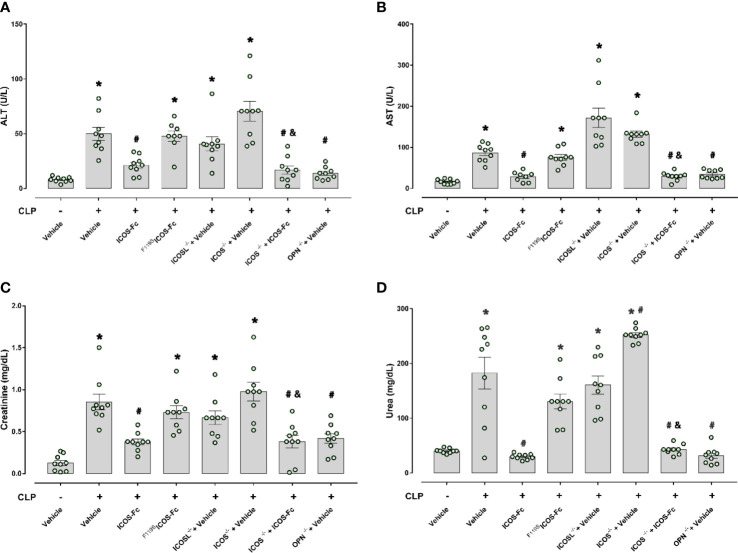
Effect of ICOS-ICOSL axis immunomodulation on sepsis-induced organ damage biomarkers. Wild-type mice and/or ICOSL, ICOS and OPN knockout mice were randomly selected to undergo either Sham or CLP surgery. One hour later, mice received once either Vehicle (PBS, 100 µL), ICOS-Fc (100 µg) or ^F119S^ICOS-Fc (100 µg) intravenously. At 24 h, blood samples were withdrawn from each mouse and plasma levels of alanine transaminase (ALT) **(A)**, aspartate transaminase (AST) **(B)**, creatinine **(C)** and urea **(D)** were determined. Data are expressed as dot plots (for each animal) and as mean ± S.E.M of 9 mice per group. Statistical analysis was performed by one-way ANOVA followed by Bonferroni’s *post hoc* test. *p<0.05 *vs* Sham + Vehicle; ^#^p<0.05 *vs* CLP + Vehicle; ^&^p<0.05 *vs* ICOS^-/-^ + Vehicle.

To investigate organ injury or dysfunction, plasma levels of ALT, AST, creatinine and urea were evaluated in these mice. **Figure 3** shows that results mirrored those shown in Fig.2: CLP-induced sepsis caused striking increase of ALT, AST, creatinine and urea levels in WT type mice, and these levels were decreased by treatment with ICOS-Fc, but not ^F119S^ICOS-Fc. Levels of these markers were increased also in CLP ICOS^-/-^ and ICOSL^-/-^ mice and urea levels were even higher in ICOS^-/-^ than in WT mice. In CLP ICOS^-/-^ mice, treatment with ICOS-Fc significantly decreased all these markers. In CLP OPN^-/-^ mice, levels of these markers were significantly lower than in CLP WT mice.

### ICOS-Fc administration modulates experimental sepsis-induced cytokine storm

**Figure 4:**
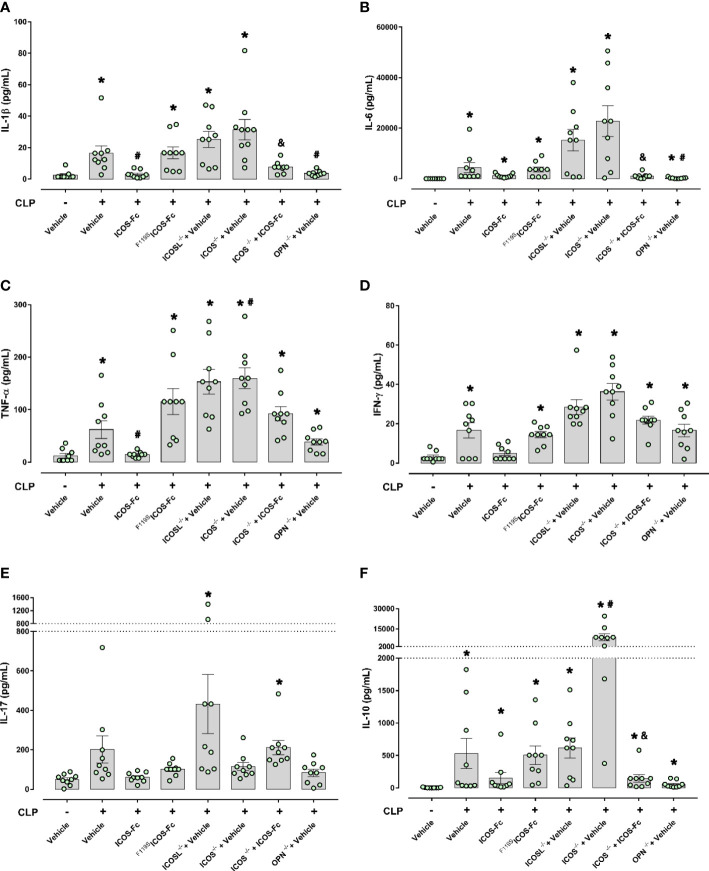
Effect of ICOS-ICOSL axis immunomodulation on systemic cytokines during experimental sepsis. Wild-type mice and/or ICOSL, ICOS and OPN knockout mice were randomly selected to undergo either Sham or CLP surgery. One hour later, mice received once either Vehicle (PBS, 100 µL), ICOS-Fc (100 µg) or ^F119S^ICOS-Fc (100 µg) intravenously. At 24 h, blood samples were withdrawn from each mouse and plasma levels of IL-1β **(A)**, IL-6 **(B)**, TNF-α **(C)**, IFN-γ **(D)**, IL-17 **(E)** and IL-10 **(F)** were determined. Data are expressed as dot plots (for each animal) and as mean ± S.E.M of 9 mice per group. Statistical analysis was performed by one-way ANOVA followed by Bonferroni’s *post hoc* test. *p<0.05 *vs* Sham + Vehicle; ^#^p<0.05 *vs* CLP + Vehicle; ^&^p<0.05 *vs* ICOS^-/-^ + Vehicle.

The 6 cytokines were measured systemically in plasma samples by using a multiplex array. **Figure 4** shows that, in WT mice, CLP-induced sepsis led to a cytokine storm with significant increase of levels of IL-1β, IL-6, IL-10, TNF-α, IFN-γ and a slight not significant increase of IL-17 compared to Sham mice. Administration of ICOS-Fc to WT CLP mice induced a significant decrease of IL-1β and TNF-α, whereas ^F119S^ICOS-Fc had no effect. Levels of IL-1β, IL-6, IL-10, TNF-α, and IFN-γ were also increased in CLP ICOS^-/-^ and ICOSL^-/-^ mice at levels similar to those observed in CLP WT mice. Moreover, CLP ICOSL^-/-^ mice showed higher levels of IL-17 than Sham mice, and CLP ICOS^-/-^ mice displayed higher levels of TNF-α and, especially, IL-10 than CLP WT mice. The CLP ICOS^-/-^ mice treated with ICOS-Fc significantly decreased levels of IL-1β, IL-6 and IL-10 compared to the untreated counterparts. In CLP OPN^-/-^ mice, the increase of these cytokines was in general moderate, with levels of IL-6, IL-10, TNF-α and IFN-γ higher than in Sham mice, and levels of IL-1β and IL-6 lower than in CLP WT mice.

### ICOS-Fc treatment reduces sepsis-induced increase in MPO activity in the kidney

**Figure 5:**
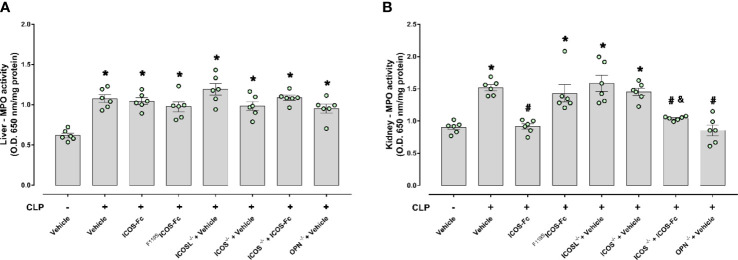
Effect of ICOS-ICOSL axis immunomodulation on sepsis-induced neutrophil (MPO activity) infiltration. Wild-type mice and/or ICOSL, ICOS and OPN knockout mice were randomly selected to undergo either Sham or CLP surgery. One hour later, mice received once either Vehicle (PBS, 100 µL), ICOS-Fc (100 µg) or ^F119S^ICOS-Fc (100 µg) intravenously. At 24 h, liver and kidney samples were harvested. Through an *in vitro* assay, myeloperoxidase (MPO) activity was measured in liver **(A)** and kidney **(B)**. Data are expressed as dot plots (for each animal) and as mean ± S.E.M of 6 mice per group. Statistical analysis was performed by one-way ANOVA followed by Bonferroni’s *post hoc* test. *p<0.05 *vs* Sham + Vehicle; ^#^p<0.05 *vs* CLP + Vehicle; ^&^p<0.05 *vs* ICOS^-/-^ + Vehicle.

MPO activity was assessed in the liver and kidney, as an indirect biomarker of leukocyte tissue infiltration (**Figure 5**). When compared to Sham mice, CLP WT mice had increased MPO activity in both liver and kidney samples, and MPO activity was significanly decreased by ICOS-Fc (but not ^F119S^ICOS-Fc treatment) in the kidney, but not in the liver. In the liver, MPO activity was similarly increased also in CLP ICOS^-/-^, ICOSL^-/-^, and OPN^-/-^ mice, and it was not modified by ICOS-Fc treatment in CLP ICOS^-/-^ mice. In the kidney, MPO activity was increased in CLP ICOS^-/-^ and ICOSL^-/-^ mice, and treatment with ICOS-Fc decreased MPO activity in CLP ICOS^-/-^ mice. By contrast, CLP OPN^-/-^ mice showed lower MPO levels in the kidney than CLP WT mice.

### ICOS-Fc treatment reduces local FAK/p38 signalling and NLRP3 inflammasome activation in septic mice

**Figure 6:**
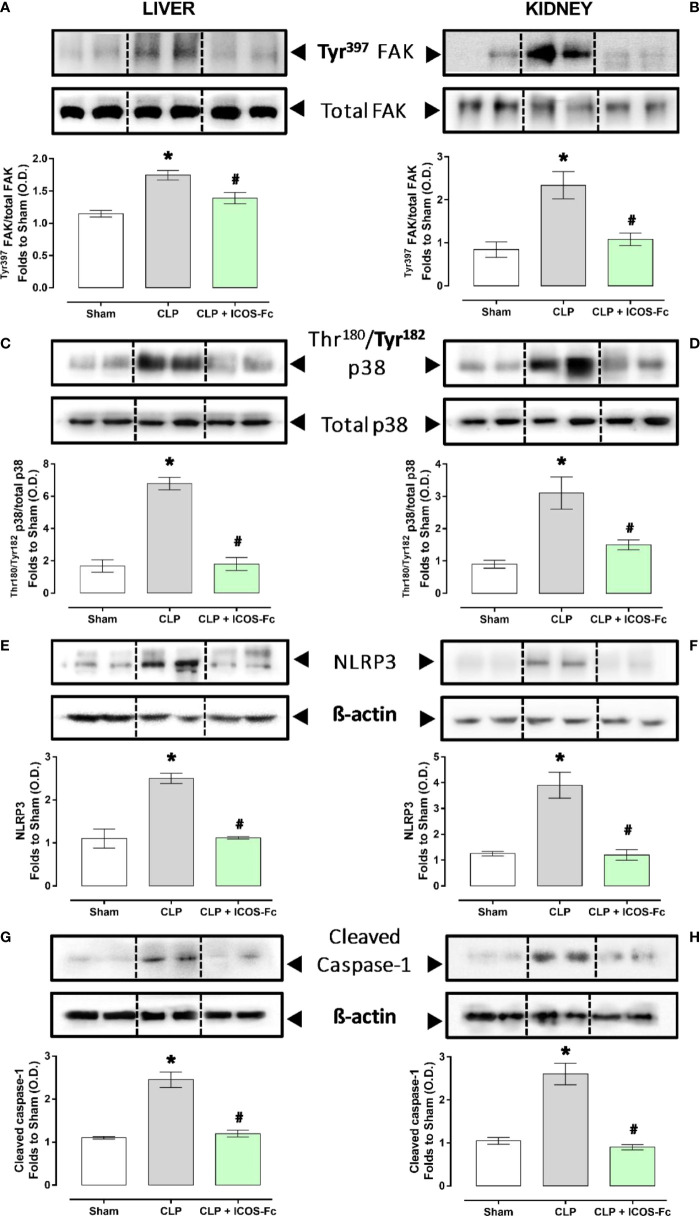
Effect of ICOS-ICOSL axis immunomodulation on tissue inflammatory pathways during experimental sepsis. Wild-type mice and/or ICOSL, ICOS and OPN knockout mice were randomly selected to undergo either Sham or CLP surgery. One hour later, mice received once either Vehicle (PBS, 100 µL), ICOS-Fc (100 µg) or ^F119S^ICOS-Fc (100 µg) intravenously. At 24 h, liver and kidney samples were harvested, and total proteins were extracted from them. Western blotting analysis for phosphorylation of Tyr^397^ on FAK in the liver **(A)** and kidney **(B)** were normalized to total FAK; Phosphorylation of Thr^180^/Tyr^182^ on p38 in the liver **(C)** and kidney **(D)** were normalized to total p38; NLRP3 expression in the liver **(E)** and kidney **(F)** were corrected against β-actin and normalized using the Sham related bands; Cleaved caspase-1 expression in the liver **(G)** and kidney **(H)** were corrected against β-actin and normalized using the Sham related bands. Densitometric analysis of the bands are expressed as relative optical density (O.D.). Data are expressed as dot plots (for each animal) and as mean ± S.E.M of 4-5 mice per group. Statistical analysis was performed by one-way ANOVA followed by Bonferroni’s *post hoc* test. *p<0.05 *vs* Sham + Vehicle; ^#^p<0.05 *vs* CLP + Vehicle.

In order to better elucidate the molecular mechanism underlying the beneficial effects evoked by ICOS-Fc administration, we focused on WT mice investigating the changes in some signaling cascades, previously documented to be affected by the ICOS-ICOSL axis and, at the same time, known to exert key role in sepsis pathogenesis. Western blot analysis showed that CLP mice showed significant increase of the phosphorylation of FAK at Tyr^397^ and p38 MAPK at Thr^180^/Tyr^182^ in both hepatic (**Figures 6A, C**) and renal (**Figures 6B, D**) tissues, when compared to Sham mice. Interestingly, mice treatment with ICOS-Fc significantly attenuated the degree of phosphorylation of FAK/p38 axis in both tissues, thus suggesting reduced activation of these signaling pathways (**Figures 6A–D**).

We then assessed the activation of the inflammasome, by evaluating the expression of NLRP3 and cleaved caspase-1 in both liver and kidney samples (**Figures 6E–H**). Results showed that, in both tissues, CLP-induced sepsis significantly increased both molecules, and the increase was inhibited by mice treatment with ICOS-Fc (**Figures 6E–H**).

## Discussion

Currently, most research on sepsis is focused on blocking the initial hyperinflammation, which in turn has resulted in promising outcomes. However, recent reports showed that both pro- and anti-inflammatory responses occur immediately and simultaneously after the onset of sepsis and most patients who survive this initial hyperinflammatory phase develop an immunosuppressive phase that can progress to late deaths (1, 32 and 33). Among the main causes of death in this immunosuppressive phase, the failure to control a primary infection and/or secondary hospital-acquired infections stands out (34). In the present study we report for the first time that ICOS-ICOSL axis may play a role in regulation of uncontrolled inflammation and organ injury induced by sepsis and that treatment of septic mice with ICOS-Fc may represent a novel immunomodulatory pharmacological approach that can simultaneously counteract both sepsis-induced hyperinflammation and immunosuppression.

These findings were obtained by evoking polymicrobial sepsis in either WT mice and knockout mice for ICOS, ICOSL and OPN genes. As expected, severe sepsis (score ≥3) was observed in vehicle-treated septic mice, suggesting potential late deaths, since the clinical scoring system is used as a surrogate marker of mortality. This detrimental effect was also associated with low body temperature (~27°C), as similarly, hypothermia is another surrogate marker of mortality, as a 5°C decrease over time or <30°C has also been shown to predict death in CLP-induced septic mice (35). Moreover, septic mice showed liver and kidney damage, displayed by increase of plasma AST/ALT and creatinine/urea levels, respectively, which is in line with the notion that sepsis can cause multiple organ failure including hepatocellular injury and renal dysfunction.

Intriguingly, treatment with ICOS-Fc substantially ameliorated the clinical picture by significantly decreasing all these parameters of sepsis. The effect was specific since no protection was detected following administration of ^F119S^ICOS-Fc (a mutated form of ICOS-Fc carrying a phenylalanine-to-serine substitution at position 119).

Theoretically, the protective activity of ICOS-Fc might be ascribed to a twofold mechanism, i.e. on the one hand to the inhibition of the endogenous ICOS activity and, on the other hand, to triggering of the endogenous ICOSL. However, the effectiveness of ICOS-Fc not only in WT mice but also in ICOS^-/-^ mice, lacking the endogenous ICOS, strongly suggest that the main protective effect on sepsis is due to triggering of ICOSL, which is in line with previous works showing that ICOSL triggering by ICOS-Fc elicits several anti-inflammatory activities both *in vitro* and *in vivo* (13, 15, 16, 19).

These results are in keeping also with recent findings showing that ICOS-Fc protects against liver damage through a shift of pro-inflammatory monocyte-derived macrophages to an anti-inflammatory phenotype (20). In parallel, the direct renoprotective effect triggered by ICOS-Fc treatment is supported by a recent study showing a key role of ICOSL in preventing early kidney disease, possibly through a selective binding to podocyte αvβ3 integrin, in which ICOSL serves as an αvβ3-selective antagonist that maintains adequate glomerular filtration (36).

The use of knockout mice highlighted that, in sepsis, a key role may be played by OPN as all the above septic parameters were significantly decreased in OPN^-/-^ mice, so that OPN deficiency mirrored the effect of ICOS-Fc in WT mice. This finding is in line with data showing that, in humans, OPN levels are increased in sepsis (37) and OPN might be involved in the sepsis pathogenesis, possibly by supporting IL-6 secretion (38). Moreover, several reports showed that ICOS-Fc inhibits several proinflammatory activities of OPN *in vitro* and *in vivo* (16, 37, 39 and 40). Our findings are in keeping also with recent data showing that macrophage-derived OPN promotes glomerular injury in an experimental model of inflammatory and progressive kidney disease (41). OPN is an heavily phosphorylated extracellular protein, expressed and secreted by several cell types, including macrophages, endothelial cells, dendritic cells and T-cells. It can act as a cytokine mediating several biological functions, including cell migration, adhesion, activation of inflammatory cells, and modulation of T cell activation supporting differentiation of proinflammatory type 1 (Th1) and type 17 (Th17) Th cells (42).

Analysis of plasmatic cytokines showed that, in all mouse strains, sepsis was accompanied by increase of IL-1β, IL-6, IL-10, TNF-α and IFN-γ. Moreover, increase of TNF-α and, especially, IL-10 was particularly striking in ICOS^-/-^ mice, which may point out that ICOS deficiency causes a dysregulation of activation of M1 and M2 macrophages. However, treatment with ICOS-Fc significantly decreased IL-1β and TNF-α in WT mice and IL-1β, IL-6 and IL-10 in ICOS^-/-^ mice indicating that ICOS-Fc substantially downmodulates the cytokine storm in sepsis. In OPN^-/-^ mice, increase of these cytokines was in general moderate, with a significant decrease of IL-1β and IL-6, in line with the mild sepsis developed by these mice.

**Figure 7:**
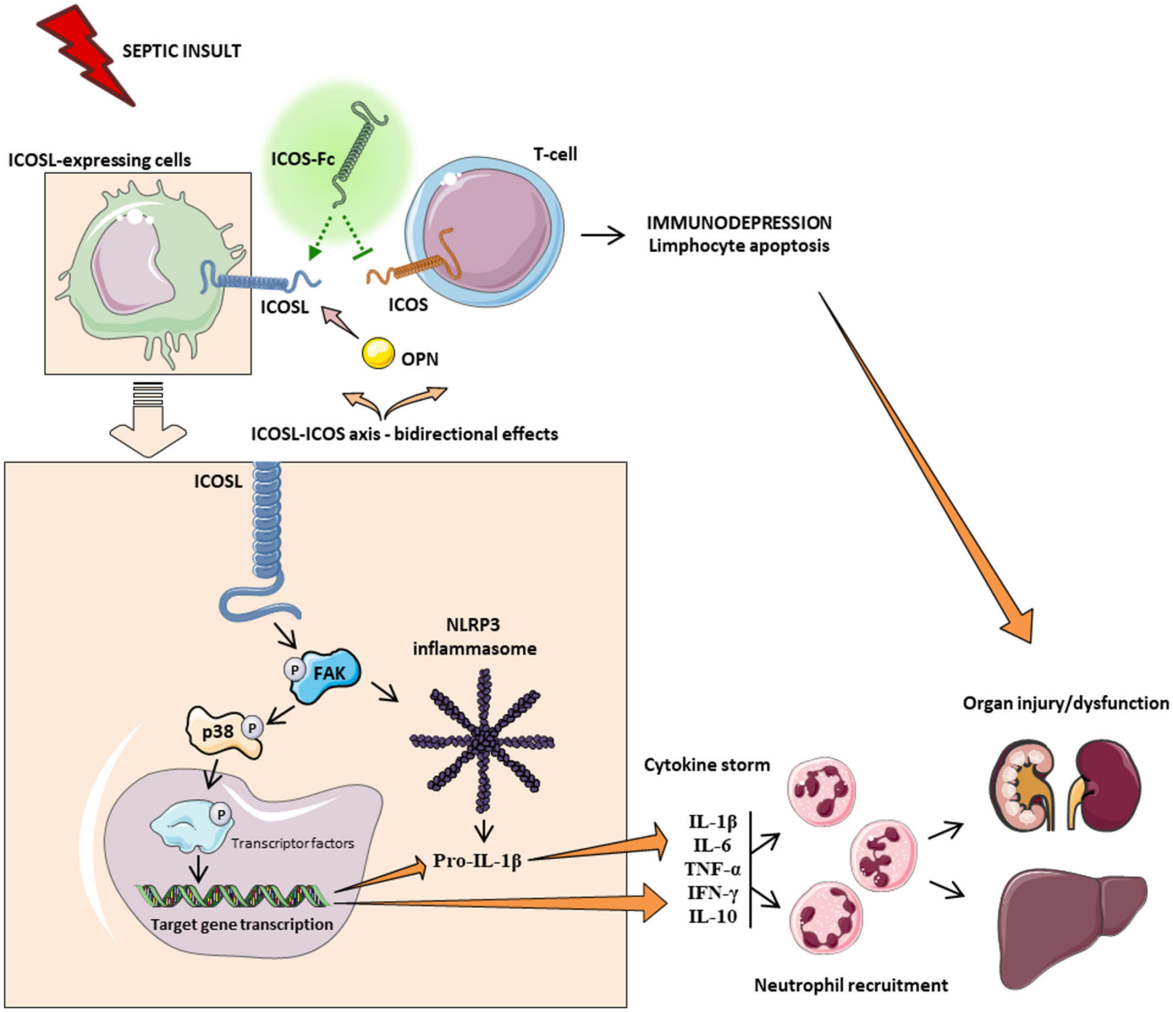
Schematic representation on the role of ICOS-ICOSL axis in the pathogenesis of sepsis. Septic insult results in an imbalance in the ICOS-ICOSL axis, leading to bidirectional harmful effects, where, on the one hand, the triggering of ICOS can induce immunosuppression, while, on the other hand, the signaling pathway downstream of the ICOSL protein leads to overactivation of FAK-p38-NLRP3 axis, promoting the transcription of pro-inflammatory genes, as well as the cleavage of pro IL-1β into IL-1β and subsequent production of pro-inflammatory cytokines. Leukocyte recruitment is also stimulated by the release of cytokines. Systemic hyperinflammation (cytokine storm), along with polymorphonuclear cell recruitment, contributes to the onset of multiple organ failure. Treatment with ICOS-Fc can attenuate sepsis-induced hyperinflammation and therefore MOF to improve clinical outcomes.

Among the main inflammatory pathways activated during sepsis, we report a local (liver and kidney) overactivation of the FAK and p38 MAPK pathways in CLP mice. Previously, we have shown that the FAK pathway mediates inflammation through p38 MAPK and that this inflammatory axis plays a role in exacerbating inflammation (28). Activation of this axis promotes increased expression/secretion of pro-inflammatory cytokines such as TNF-α, IL-6, IL-1β and IL-17, which in turn contribute to the cytokine storm and multiple organ failure (MOF) associated with sepsis (43). Intriguingly, treatment of septic mice with ICOS-Fc significantly attenuated FAK and p38 MAPK phosphorylation, thus reducing their activation during septic insult, with a following impact on the development of the above-mentioned cytokine storm. These findings are in accordance with previous studies focused on tumor cell migration, whose treatment with ICOS-Fc reduces FAK and p38 MAPK activation both *in vitro* and *in vivo* (15, 19). As we and other have recently shown, FAK activation may also affect the overexpression and activation of another peculiar inflammatory pathway, NLRP3 inflammasome complex (28, 44). Thus, we wondered here whether ICOS-Fc could also infer with this cross-talk mechanism linking FAK to NLRP3 activation within the septic context. We report here that experimental sepsis led to an overactivation of the NLRP3 complex and consequent activation of its downstream mediator caspase-1, which were significantly reduced by treatment with ICOS-Fc, thus leading to reduced systemic release of IL-1β. In addition to the impact on the aforementioned inflammatory pathways, ICOS-Fc administration seems to directly affect leukocyte migration in CLP mice, as documented by the changes in MPO activity, a well-known biomarker of neutrophil infiltration, in both liver and kidney homogenates (45). Specifically, we documented that the sepsis-induced increase in MPO activity in renal tissues, was significantly counteracted by ICOS-Fc treatment. This effect, on the other hand, was absent when CLP mice were treated with ^F119S^ICOS-Fc. Intriguingly, increased MPO activity was recorded in liver homogenates from septic mice, regardless of drug treatment or genetic intervention, when compared to Sham mice. Despite ICOS-Fc has been shown to reduce the migration of polymorphonuclear cells into inflamed tissues (15), these discrepant events observed in liver and kidney tissue may be the result of different levels of ICOSL expression. This finding corroborates a previous study reporting that hepatocytes did not express ICOSL, when compare to other organs, such as the kidney (46). Thus, suggesting that the hepatic protection induced by ICOS-Fc in septic mice is mainly due to a local and systemic resolution of inflammation rather than a reduction in leukocyte infiltration. A schematic representation summarizing the role of ICOS-ICOSL axis in the pathogenesis of sepsis and the protective effects of ICOS-Fc following sepsis-induced multiple organ failure is shown in **Figure 7**.

Despite the originality of our findings, we are aware of several limitations of our study, including the lack of extension of these findings to other important functional organs related to MOF during sepsis, such as the lungs and the cardiac tissue, along with the lack of analysis suggestive of the direct effect of ICOS-Fc treatment in preventing immunosuppression. Albeit the *in vivo* protocol described here is in accordance with the main recommendations provided by MQTiPSS consensus guidelines (27), we are not authorized to perform a survival study to assess the long-term effect of ICOS-Fc due to ethical reasons. Thus, further studies are needed to extend the clinical relevance of our findings as well as to gain a better insight into the safety profile of the proposed drug treatment.

## Conclusions

In conclusion, we demonstrate here, for the first time, that the ICOS-ICOSL axis plays a crucial role in the development of systemic inflammation and organ damage induced by a clinically relevant sepsis model. These findings were confirmed by an exacerbation of septic injury in mice knockout for the ICOS and ICOSL genes. Interestingly, we also documented its draggability by showing protection when ICOS-Fc, a recombinant protein which act as an antagonist of ICOS and an agonist of ICOSL, was administered during sepsis. The beneficial effects of this innovative pharmacological approach are likely due to a potential cross-talk mechanisms involving the FAK-p38-NLRP3 inflammasome axis. A greater understanding of the molecular basis of ICOS-Fc-mediated effects is needed to harness its actions as a potentially powerful immunomodulatory tool for counteracting inflammation and organ injury in sepsis.

## Data availability statement

The raw data supporting the conclusions of this article will be made available by the authors, without undue reservation.

## Ethics statement

The animal study was reviewed and approved by Ethical committee OPBA University of Turin and Italian Ministry of Health, Italy.

## Author contributions

GA, CD, UD, and MC conceived and designed the experiments. GA, IS, EA, CM, RM, EP, GE, DC, and NC performed the experiments. GA, EA, CM, RM, IB, EB, CG, NC, MA, DF, CT, CC, CD, UD, and MC analyzed the data. GA, CD, UD, CT, and MC, writing - review and editing. All authors have read and agreed to the published version of the manuscript.

## Funding

The Università degli Studi di Torino has supported and funded this work (Ricerca Locale 2020 and 2021) and by the Associazione Italiana Ricerca sul Cancro (IG20714), Milan Italy.

## Acknowledgments

Parts of the **Figure 7** were drawn by using pictures from Servier Medical Art. Servier Medical Art by Servier is licensed under a Creative Commons Attribution 3.0 Unported License (https://creativecommons.org/licenses/by/3.0/).

## Conflict of interest

The authors declare that the research was conducted in the absence of any commercial or financial relationships that could be construed as a potential conflict of interest.

## Publisher’s note

All claims expressed in this article are solely those of the authors and do not necessarily represent those of their affiliated organizations, or those of the publisher, the editors and the reviewers. Any product that may be evaluated in this article, or claim that may be made by its manufacturer, is not guaranteed or endorsed by the publisher.

## References

[1] Hotchkiss RS Monneret G Payen D. Sepsis-induced immunosuppression: From cellular dysfunctions to immunotherapy. Nat Rev Immunol (2013) 13:862–74. doi: 10.1038/nri3552 PMC4077177 24232462

[2] Rudd KE Johnson SC Agesa KM Shackelford KA Tsoi D Kievlan DR. Global, regional, and national sepsis incidence and mortality, 1990 – 2017 : analysis for the global burden of disease study. Lancet (2020) 395:200–11. doi: 10.1016/S0140-6736(19)32989-7 PMC6970225 31954465

[3] Hutloff A Dittrich AM Beier KC Eljaschewitsch B Kraft R Anagnostopoulos I. ICOS is an inducible T-cell co-stimulator structurally and functionally related to CD28. Nature (1999) 100:263–6. doi: 10.1038/16717 9930702

[4] Yoshinaga SK Whoriskey JS Khare SD Sarmiento U Guo J Horan T. T-Cell co-stimulation through B7RP-1 and ICOS. Nature (1999) 1:827–32. doi: 10.1038/45582 10617205

[5] Swallow MM Wallin JJ Sha WC. B7h, a novel costimulatory homolog of B7.1 and B7.2, is induced by TNFa. Immunity (1999) 11:423–32. doi: 10.1016/s1074-7613(00)80117-x 10549624

[6] Möhnle P Hirschberger S Hinske LC Briegel J Hübner M Weis S. MicroRNAs 143 and 150 in whole blood enable detection of T-cell immunoparalysis in sepsis. Mol Med (2018) 24:1–14. doi: 10.1186/s10020-018-0056-z 30332984 PMC6191918

[7] Menéndez R Méndez R Almansa R Ortega A Alonso R Suescun M. Simultaneous depression of immunological synapse and endothelial injury is associated with organ dysfunction in community-acquired pneumonia. J Clin Med (2019) 8:1–10. doi: 10.3390/jcm8091404 PMC6780106 31500177

[8] Burmeister Y Lischke T Dahler AC Mages HW Lam K-P Coyle AJ. ICOS controls the pool size of effector-memory and regulatory T cells. J Immunol (2008) 180:774–82. doi: 10.4049/jimmunol.180.2.774 18178815

[9] Chen Q Mo L Cai X Wei L Xie Z Li H. ICOS signal facilitates Foxp3 transcription to favor suppressive function of regulatory T cells. Int J Med Sci (2018) 15:666–73. doi: 10.7150/ijms.23940 PMC6001412 29910670

[10] Luan Y Yin C Qin Q Dong N Zhu X Sheng Z. Effect of regulatory T cells on promoting apoptosis of T lymphocyte and its regulatory mechanism in sepsis. J Interf Cytokine Res (2015) 35:969–80. doi: 10.1089/jir.2014.0235 PMC4683547 26309018

[11] Saito K Wagatsuma T Toyama H Ejima Y Hoshi K Shibusawa M. Sepsis is characterized by the increases in percentages of circulating CD4 + CD25 + regulatory T cells and plasma levels of soluble CD25. J Exp Med (2008) 216:61–8. doi: 10.1620/tjem.216.61 18719339

[12] Leng F Liu J Liu Z Yin J Qu H-P. Increased proportion of CD4 d CD25 d Foxp3 d regulatory T cells during early-stage sepsis in ICU patients. J Microbiol Immunol Infect (2013) 46:338–44. doi: 10.1016/j.jmii.2012.06.012 22921804

[13] Occhipinti S Dianzani C Chiocchetti A Boggio E Clemente N Gigliotti CL. Triggering of B7h by the ICOS modulates maturation and migration of monocyte-derived dendritic cells. J Immunol (2013) 190:1125–34. doi: 10.4049/jimmunol.1201816 23275603

[14] Lund SA Giachelli CM. The role of osteopontin in inflammatory processes. J Cell Commun Signal (2009) 3:311–22. doi: 10.1007/s12079-009-0068-0 PMC2778587 19798593

[15] Dianzani C Minelli R Mesturini R Chiocchetti A Barrera G Boscolo S. B7h triggering inhibits umbilical vascular endothelial cell adhesiveness to tumor cell lines and polymorphonuclear cells. J Immunol (2010) 185:3970–9. doi: 10.4049/jimmunol.0903269 20817864

[16] Raineri D Dianzani C Cappellano G Maione F Baldanzi G Iacobucc I. Osteopontin binds ICOSL promoting tumor metastasis,”. Commun Biol (2020) 3:1–15. doi: 10.1038/s42003-020-01333-1 33106594 PMC7588454

[17] Di Niro R Ziller F Florian F Crovella S Stebel M Bestagno M. Construction of miniantibodies for the *in vivo* study of human autoimmune diseases in animal models. BMC Biotechnol (2007) 7:1–10. doi: 10.1186/1472-6750-7-46 17678525 PMC1963447

[18] Gigliotti CL Boggio E Clemente N Shivakumar Y Toth E Sblattero D. ICOS-ligand triggering impairs osteoclast differentiation and function *In vitro* and *In vivo* . J Immunol (2016) 197:3905–16. doi: 10.4049/jimmunol.1600424 27798154

[19] Dianzani C Minelli R Gigliotti CL Occhipinti S Giovarelli M Conti LM. B7h triggering inhibits the migration of tumor cell lines. J Immunol (2014) 192:4921–31. doi: 10.4049/jimmunol.1300587 24729612

[20] Ramavath NN Gadipudi LL Provera A Gigliotti LC Boggio E Bozzola C. Inducible T-cell costimulator mediates Lymphocyte/Macrophage interactions during liver repair. Front Immunol (2021) 12:786680. doi: 10.3389/fimmu.2021.786680 34925367 PMC8678521

[21] Stoppa I Gigliotti CL Clemente N Pantham D Dianzani C Monge C. ICOSL stimulation by ICOS-fc accelerates cutaneous wound healing *In vivo* . Int J Mol Sci (2022) 23:1–12. doi: 10.3390/ijms23137363 PMC9266942 35806368

[22] O’Sullivan AW Wang JH Redmond HP. NF-κB and P38 MAPK inhibition improve survival in endotoxin shock and in a cecal ligation and puncture model of sepsis in combination with antibiotic therapy. J Surg Res (2009) 152:46–53. doi: 10.1016/j.jss.2008.04.030 19027920

[23] Cornelius DC Travis OK TramelI RW Borges-Rodriguez M Baik CH Greer M. NLRP3 inflammasome inhibition attenuates sepsis-induced platelet activation and prevents multi-organ injury in cecal-ligation puncture. PLoS One (2020) 15:1–15. doi: 10.1371/journal.pone.0234039 PMC7299389 32555710

[24] Lee S Nakahira K Dalli J Siempos II Norris PC Colas RA. NLRP3 inflammasome deficiency protects against microbial sepsis *via* increased lipoxin B4 synthesis. Am J Respir Crit Care Med (2017) 196:713–26. doi: 10.1164/rccm.201604-0892OC PMC5620673 28245134

[25] Chen X Zhao Y Wang X Lin Y Zhao W Wu D. FAK mediates LPS-induced in fl ammatory lung injury through interacting TAK1 and activating TAK1-NF κ b pathway. Cell Death Differ (2022) 13:1–12. doi: 10.1038/s41419-022-05046-7 PMC9270420 35803916

[26] du Sert NP Hurst V Ahluwalia A Alam S Avey MT Baker M. The arrive guidelines 2.0: Updated guidelines for reporting animal research. PloS Biol (2020) 18:1–12. doi: 10.1371/journal.pbio.3000410 PMC7360023 32663219

[27] Osuchowski MF Ayala A Bahrami S Bauer M Boros M Cavaillon J-M. Minimum quality threshold in pre-clinical sepsis studies (mqtipss): An international expert consensus initiative for improvement of animal modeling in sepsis. Shock (2018) 50:377–80. doi: 10.1097/SHK.0000000000001212 PMC6133201 30106875

[28] Alves GF Aimaretti E Einaudi G Mastrocola R Oliveira JG Collotta D. Pharmacological inhibition of FAK-Pyk2 pathway protects against organ damage and prolongs the survival of septic mice. Front Immunol (2022) 13:837180. doi: 10.3389/fimmu.2022.837180 35178052 PMC8843946

[29] Kovalski V Prestes AP Oliveira JG Alves GF Colarites D El Mattos J. Protective role of cGMP in early sepsis. Eur J Pharmacol (2017) 807:174–81. doi: 10.1016/j.ejphar.2017.05.012 28483456

[30] Nandra KK Collino M Rogazzo M Fantozzi R Patel NSA Thiemermann C. “Pharmacological preconditioning with erythropoietin attenuates the organ injury and dysfunction induced in a rat model of hemorrhagic shock,”. DMM Dis Model Mech (2013) 6:701–9. doi: 10.1242/dmm.011353 PMC3634653 23264564

[31] Faul F Erdfelder E Lang A-G Buchner A. G*Power 3: A flexible statistical power analysis program for the social, behavioral, and biomedical sciences. Behav Res Methods (2007) 39:175–91. doi: 10.3758/bf03193146 17695343

[32] Tsirigotis P Chondropoulos S Gkirkas K Meletiadis J Dimopoulou I. “Balanced control of both hyper and hypo-inflammatory phases as a new treatment paradigm in sepsis,”. J Thorac Dis (2016) 8:E312–6. doi: 10.21037/jtd.2016.03.47 PMC4842841 27162689

[33] Boomer JS To K Chang KC Takasu O Osborne DF Walton AH. Immunosuppression in patients who die of sepsis and multiple organ failure. J Am Med Assoc (2011) 306:2594–605. doi: 10.1001/jama.2011.1829 PMC3361243 22187279

[34] Otto GP Sossdorf M Claus RA Rödel J Menge K Reinhart K. The late phase of sepsis is characterized by an increased microbiological burden and death rate. Crit Care (2011) 15:1–8. doi: 10.1186/cc10332 PMC3387626 21798063

[35] Mai SHC Sharma N Kwong AC Dwivedi DJ Khan M Grin PM. Body temperature and mouse scoring systems as surrogate markers of death in cecal ligation and puncture sepsis. Intensive Care Med Exp (2018) 6:1–14. doi: 10.1186/s40635-018-0184-3 PMC6063809 30054760

[36] Koh KH Cao Y Mangos S Tardi NJ Dande RR Lee HW. Nonimmune cell–derived ICOS ligand functions as a renoprotective αvβ3 integrin–selective antagonist. J Clin Invest (2019) 129:1713–26. doi: 10.1172/JCI123386 PMC6436851 30747722

[37] Castello LM Baldrighi M Molinari L Salmi L Cantaluppi V Vaschetto R. The role of osteopontin as a diagnostic and prognostic biomarker in sepsis and septic shock. Cells (2019) 8:1–12. doi: 10.3390/cells8020174 PMC6407102 30781721

[38] Uchibori T Matsuda K Shimodaira T Sugano M Uehara T Honda T. IL-6 trans-signaling is another pathway to upregulate osteopontin. Cytokine (2017) 90:88–95. doi: 10.1016/j.cyto.2016.11.006 27863335

[39] Hirano Y Aziz M Yang W-L Wang Z Zhou M Ochani M. Neutralization of osteopontin attenuates neutrophil migration in sepsis-induced acute lung injury. Crit Care (2015) 19:1–15. doi: 10.1186/s13054-015-0782-3 25887405 PMC4345018

[40] Fortis S Khadaroo RG Haitsma JJ Zhang H. Osteopontin is associated with inflammation and mortality in a mouse model of polymicrobial sepsis. Acta Anaesthesiol Scand (2015) 59:170–5. doi: 10.1111/aas.12422 PMC4936904 25328143

[41] Trostel J Truong LD Roncal-Jimenez C Miyazaki M Miyazaki-Anzai S Kuwabara M. Disease different effects of global osteopontin and macrophage osteopontin in glomerular injury. Am J Physiol - Ren Physiol (2018) 315:F759–68. doi: 10.1152/ajprenal.00458.2017 PMC6230752 29717936

[42] Boggio E Dianzani C Gigliotti CL Soluri MF Clemente N Cappellano G. Thrombin cleavage of osteopontin modulates its activities in human cells *in vitro* and mouse experimental autoimmune encephalomyelitis *in vivo* . J Immunol Res (2016) 2016:1–13. doi: 10.1155/2016/9345495 PMC4961817 27478856

[43] Chaudhry H Zhou J Zhong Y Ali MM Mcguire F Nagarkatti PS. Role of cytokines as a double-edged sword in sepsis. In Vivo (Brooklyn) (2013) 27:669–84. PMC4378830 24292568

[44] Chung IC OuYang C-N Yuan S-N Li H-P Chen J-T Shieh H-R. Pyk2 activates the NLRP3 inflammasome by directly phosphorylating ASC and contributes to inflammasome-dependent peritonitis. Sci Rep (2016) 6:1–13. doi: 10.1038/srep36214 27796369 PMC5087076

[45] Yu H Liu Y Wang M Restrepo RJ Wang D Kalogeris TJ. Myeloperoxidase instigates proinflammatory responses in a cecal ligation and puncture rat model of sepsis. Am J Physiol - Hear Circ Physiol (2020) 319:H705–21. doi: 10.1152/ajpheart.00440.2020 PMC7509276 32762560

[46] Wahl C Bochtler P Chen L Schirmbeck R Reimann J. B7-H1 on hepatocytes facilitates priming of specific cd8 t cells but limits the specific recall of primed responses. Gastroenterology (2008) 135:980–8. doi: 10.1053/j.gastro.2008.05.076 18621049

